# When Characteristics of Clinical Trials Require Per-Protocol as Well as Intention-to-Treat Outcomes to Draw Reliable Conclusions: Three Examples

**DOI:** 10.3390/jcm12113625

**Published:** 2023-05-23

**Authors:** David E. Scheim, Colleen Aldous, Barbara Osimani, Edmund J. Fordham, Wendy E. Hoy

**Affiliations:** 1 US Public Health Service, Commissioned Corps, Inactive Reserve, Blacksburg, VA 24060, USA; 2 College of Health Sciences, University of KwaZulu-Natal, Durban 4041, South Africa; 3 Center for Philosophy, Science, and Policy, Faculty of Medicine, Marche Polytechnic University, 60121 Ancona, Italy; 4 EbMCsquared CIC, Bath BA2 4BL, UK; 5 Centre of Chronic Disease, Faculty of Medicine, University of Queensland, Brisbane 4072, Australia

**Keywords:** intention-to-treat, per-protocol, colonoscopy, fluvoxamine, ivermectin, COVID-19

## Abstract

Under exceptional circumstances, including high rates of protocol non-compliance, per-protocol (PP) analysis can better indicate the real-world benefits of a medical intervention than intention-to-treat (ITT) analysis. Exemplifying this, the first randomized clinical trial (RCT) considered found that colonoscopy screenings were marginally beneficial, based upon ITT analysis, with only 42% of the intervention group actually undergoing the procedure. However, the study authors themselves concluded that the medical efficacy of that screening was a 50% reduction in colorectal cancer deaths among that 42% PP group. The second RCT found a ten-fold reduction in mortality for a COVID-19 treatment drug vs. placebo by PP analysis, but only a minor benefit by ITT analysis. The third RCT, conducted as an arm of the same platform trial as the second RCT, tested another COVID-19 treatment drug and reported no significant benefit by ITT analysis. Inconsistencies and irregularities in the reporting of protocol compliance for this study required consideration of PP outcomes for deaths and hospitalizations, yet the study coauthors refused to disclose them, instead directing inquiring scientists to a data repository which never held the study’s data. These three RCTs illustrate conditions under which PP outcomes may differ significantly from ITT outcomes and the need for data transparency when these reported or indicated discrepancies arise.

## 1. Introduction

For randomized clinical trials (RCTs) that evaluate drug efficacy, intention-to-treat (ITT) analysis, which considers outcomes for all subjects in the treatment and placebo groups regardless of protocol compliance, is generally deemed most reliable [1,2]. However, under certain circumstances, for example, when protocol compliance is low, ITT analysis can have large negative biases [1,3,4] and can underestimate the efficacy of a novel treatment [5]. In such cases, per-protocol (PP) outcomes must be considered as well, and may provide a more reliable measure of therapeutic efficacy. For the first two RCTs of this examination, PP and ITT analyses yielded significantly different conclusions, and for each, the respective coauthors found that its PP analysis likely assessed the medical efficacy of its intervention. For the third RCT considered, inconsistencies and irregularities in the reporting of protocol compliance for its placebo group, as well as a higher protocol non-compliance rate than that of the second RCT, necessitated disclosure of PP outcomes, but these were not provided, despite repeated requests. We will use these three examples to indicate conditions under which PP outcomes must be considered in order to draw reliable conclusions, and also conditions under which PP analysis is more likely than ITT analysis to be predictive of real-world outcomes.

## 2. Three RCTs for Which PP Outcomes Were Needed to Draw Reliable Conclusions

### 2.1. Colonoscopy Screenings Were Found Effective, but Only When Actually Performed

The first RCT of our focus, Bretthauer et al. 2022 [6], studied the preventative efficacy of colonoscopy screenings for colorectal cancer (CRC) incidence and deaths in subjects from four European countries over a ten-year period. Based upon the ITT analysis of comparative outcomes for the intervention group, consisting of subjects invited to undergo this screening, and control subjects who did not undergo this screening, relative risks (RRs) of CRC incidence and deaths of 0.82 and 0.90, respectively, were reported. An editorial published in conjunction with this study [7] characterized these relatively minor reported benefits from colonoscopy screening as “surprising and disappointing,” a conclusion that was echoed in news articles [8,9].

Yet a critical flaw of this ITT analysis was that only 42% of the intervention group, the subjects invited to have a colonoscopy, actually underwent this procedure. Bretthauer et al. reported that in the adjusted PP analysis to estimate the benefits among those who actually had that screening, CRC deaths were reduced by half (RR = 0.50), with RR = 0.69 for CRC incidence. The problem with drawing conclusions from ITT outcomes when compliance rates are low was noted in an influential analysis by Hernan and Robins [3]. They considered a prior sigmoidoscopy screening study, Holme et al. 2014 [10], which had concluded from ITT analysis that screening yielded no significant reduction in all-cause mortality. Hernan and Robins noted, however, that this conclusion substantially underestimated the effect of the screening because only two-thirds of the intervention group actually underwent the procedure (see Appendix C, item 1).

Yet Holme et al.’s main takeaway was that its screening provided significant reductions in both CRC incidence and mortality, with that ITT all-cause mortality finding mentioned only peripherally. Bretthauer et al., in contrast, selectively emphasized the study’s ITT outcomes, which indicated minor benefits of colonoscopy screenings, including only those results in its abstract. This abstract also reported ITT outcomes for all-cause mortality, which showed no reduction in the ITT intervention group vs. controls, consistent with the similar small reduction in CRC deaths by the ITT analysis, which was well within overlapping confidence intervals. No PP outcomes were provided for all-cause mortality, and the study’s other PP outcomes, which showed, in contrast, major reductions in CRC incidence and deaths, were first mentioned far into the publication.

Although ITT analysis is subject to large negative biases when protocol compliance is low, PP analysis is subject to distortions as well, including the so-called “healthy adherers” bias [11]. Yet Bretthauer et al. themselves concluded that their study’s PP outcomes of a 31% decrease in CRC incidence and a 50% decrease in CRC deaths “probably underestimated the benefit” of the colonoscopy screening procedure. They added that “optimism” as to these reductions in CRC deaths “may be warranted in light of the 50% decrease observed in adjusted per-protocol analyses.” They explained, however, that their study mimicked a population screening program in practice, and their overall aim was “to quantify the benefits of colonoscopy in population screening.” Thus, Bretthauer et al. concluded that the PP outcome of a 50% decrease in CRC deaths likely characterized the medical efficacy of colonoscopy screenings, yet the abstract of their publication and the accompanying editorial emphasized the study’s ITT outcomes.

Bretthauer et al.’s justification for highlighting the study’s ITT results is putting the cart before the horse. Compliance with a colonoscopy screening program, aside from dependence upon non-medical factors such as effective performance by administrative staff, would likely be increased substantially by communicating to subjects that the screening cuts CRC deaths in half. Conversely, compliance would be decreased by the mistaken impression from the Bretthauer et al. abstract, its accompanying editorial, and follow-up news articles, that this screening provided minimal benefits.

### 2.2. PP and ITT Analyses Provided Divergent Indications of Drug Efficacy

Between April 2021 and March 2022, four publications reported results for an assortment of repurposed drugs tested in Brazil for COVID-19 treatment in the TOGETHER platform trial [12,13,14,15]. Both the FDA and the NIH concluded that the primary outcome reported by all arms of the TOGETHER trial, emergency room visits plus hospitalizations, was inadequate [16,17]. The FDA noted that deaths and hospitalizations, instead, were the TOGETHER trial’s most important outcomes [16], and these will be the focus of our examination for the next two RCTs considered.

The second RCT of our focus is the fluvoxamine (FLV) arm of the TOGETHER trial, henceforth designated TOGETHER-FLV [13]. It reported a statistically insignificant reduction in deaths with FLV treatment using ITT analysis: 17 deaths in the ITT treatment group of 741 (2.3%) vs. 25 deaths in the ITT placebo group of 756 (3.3%), yielding a relative risk (RR) of 0.69, *p* = 0.24. However, the PP outcomes were 1/548 (0.2%) for treatment deaths vs. 12/618 (1.9%) for placebo deaths—a 91% (ten-fold) reduction in deaths (RR = 0.09, *p* = 0.022). Suboptimal rates of protocol compliance in the treatment and placebo groups (74% and 82%, respectively) made these divergent outcomes from PP vs. ITT analyses possible.

A judgement as to whether the modest treatment benefits indicated by ITT analysis or the major benefits indicated by PP analysis had most real-world relevance would depend upon complex characteristics of the clinical trial data, such as reasons for protocol noncompliance, which are not considered here. However, the TOGETHER-FLV coauthors themselves concluded that its PP analysis had real-world relevance, noting that for patients adhering to the treatment protocol, FLV might have “considerable clinical benefits” against COVID-19 [13]. A senior coauthor of TOGETHER-FLV, Edward Mills, stated at an NIH-sponsored presentation on 6 August 2021, that the upshot of this study was that FLV “potentially provides a very large treatment effect” [18], a conclusion supported by the study’s PP outcomes, not its ITT outcomes (Appendix C, item 2). Furthermore, a researcher, David Boulware, who collaborated on two FLV studies [19,20] with a TOGETHER-FLV coauthor, Eric Lenze, and who himself coauthored a subsequent TOGETHER trial study [15], found the PP outcome data to be most meaningful. Boulware filed an application to the FDA for an emergency use authorization of FLV for COVID-19 treatment, following the publication of TOGETHER-FLV [21]. In that application, he “relie[d] heavily on the PP analyses that suggest a treatment effect on mortality,” as the FDA noted in a memo explaining its rejection of that application [16].

### 2.3. Nondisclosure of Needed PP Outcomes for a Third RCT

The third RCT of our focus is the TOGETHER trial RCT, which tested ivermectin (IVM) for treatment of COVID-19 [15], henceforth designated TOGETHER-IVM, and which concluded that it was ineffective. IVM is a macrocyclic lactone whose discovery and successful containment of two devastating global tropical diseases was recognized with the 2015 Nobel Prize for medicine [22]. Interest in the PP outcomes for TOGETHER-IVM was initially raised by the unusually large and inconsistently reported ITT-to-PP decrease in the study’s placebo group: 58% in Table 2 (of [15]) vs. 19% in Table 3 (of [15]), this discrepancy arising from conflicting descriptions of the study’s placebo group (Appendix C, item 3, below). These differing values of 58% and 19% for the ITT-to-PP placebo group decrease were each much larger than the 8% ITT-to-PP decrease in the study’s treatment group, which raises the question as to why subjects taking inert tablets instead of IVM tablets would have a non-compliance rate that was so much greater. It is also noteworthy that even the lower 19% figure for the study’s ITT-to-PP decrease in the placebo group is greater than the 18% decrease in the placebo group for TOGETHER-FLV, for which the treatment benefit was much greater as indicated by PP vs. ITT analysis.

As noted above, for the FDA and the NIH, the TOGETHER trial’s primary outcome was inadequate; deaths and hospitalizations were the outcomes of key importance [16,17]. As also noted above, ITT analysis can have large negative biases and underestimate the efficacy of a novel treatment when protocol compliance is low [1,3,4]. Several scientists therefore requested the PP outcomes for death and hospitalization rates, treatment vs. placebo, which were unreported, from coauthors of TOGETHER-IVM. These four numbers were requested in an email of 11 April 2022 [23], in a letter of 10 May 2022 signed by 66 scientists and physicians worldwide [24], and in four subsequent emails sent 10 May through 19 July 2022 (Appendix C, item 4).

The TOGETHER-IVM coauthors refused all six requests to disclose these four PP outcome numbers, even though these PP outcomes for deaths or hospitalizations were reported for TOGETHER-FLV [13] and for the two other TOGETHER trial publications [12,14]. In an email reply to the 10 May 2022 letter to the TOGETHER-IVM coauthors that requested these PP outcomes, it was, ironically, study coauthor Boulware, who had emphasized the importance of the PP mortality outcomes for FLV, who refused to disclose them for IVM. Instead, Boulware directed the requestors to the ICODA data repository [25], which TOGETHER-IVM’s data sharing statement (DSS) had listed as the data source, to be made available “immediately after publication” [26].

However, after two months of unsuccessful attempts to obtain that study data through ICODA’s listed email address (no reply or autoreply) and telephone number (“not currently set up to receive calls” [27]), it was finally learned that ICODA never held any TOGETHER trial data. On 7 June, an ICODA manager emailed one of the inquiring scientists, stating: “ICODA does not hold the data and we have requested that the authors update the data sharing agreement to reflect that” [28]. A month later, on 7 July 2022, the study’s DSS link was changed to designate a different repository, vivli.org, and on 14 July, a record for the TOGETHER trial (NCT04727424) appeared there. That Vivli record for the TOGETHER trial, however, listed three drugs, but not IVM, as the study’s treatment agents [26]. That Vivli listing was then changed to include IVM on 21 July [26] after three coauthors of this analysis (CA, DES, EJF) emailed the TOGETHER-IVM coauthors on 19 July about that omission ([24]; Appendix C, item 5). However, the Vivli data, even if it were to become available after an extended application and review process, would still not allow the determination of PP death and hospitalization rates, which requires the delineation of the composition of the ITT and PP placebo group, as requested from the study coauthors but not disclosed (see Section 5, below).

## 3. Potential Public Health Consequences from the Disregarding or Withholding of PP Outcomes in the Three RCTs Considered

Follow-up expert commentary on the Bretthauer et al., 2022 colonoscopy study, the first RCT of our focus, noted that its finding from PP analysis of significant efficacy of colonoscopy screening in reducing CRC deaths was of greater relevance to public health than the impact of a screening program with a low rate of participation, 42%, as assessed by ITT analysis [29]. The main conclusion reported by this 2022 study and the accompanying editorial [7], based upon ITT analysis, can thus result in reduced use of colonoscopy screening, negatively impacting public health.

For the second RCT considered, TOGETHER-FLV, the study’s PP analysis found a 91% reduction in deaths, FLV treatment vs. placebo, *p* = 0.022. Both the study paper and a follow-up submission to the FDA for emergency use authorization of FLV for COVID-19 treatment emphasized the real-world relevance of this PP finding. Although the FDA rejected this application, this study paper appropriately reported and evaluated data that supported an informed decision on the use of this drug for COVID-19 treatment.

For the third RCT considered, TOGETHER-IVM, the withheld PP outcome numbers could indicate drug efficacy, just as PP outcome numbers did for TOGETHER-FLV. If so, that result would align with most of the RCTs considered in a comprehensive 2021 review coauthored by the 2015 Nobel co-laureate for the discovery of IVM, which concluded that IVM was effective in both treatment and prevention of COVID-19 [30]. Several RCTs that reported positive findings for the efficacy of IVM for COVID-19 treatment and prevention were also cited in a 2021 review [22]. Remarkably, an August 2022 editorial which categorically declared IVM ineffective against COVID-19 [31], prominently cited in support a meta-analysis of RCTs for IVM treatment of COVID-19, published in June 2022 [32], which actually reported a two-fold reduction in deaths in its pooled IVM treatment vs. placebo groups. Specifically, the first finding presented in the results section of that meta-analysis was that the pooled (natural) log odds ratio (OR) for mortality in ten RCTs including 3,472 patients was “−0.67 (95% CI −1.20 to −0.13) with low heterogeneity” (I^2^ = 29%), which corresponds to an OR of 0.51 (see Appendix C, item 6).

In contrast to the positive findings for IVM treatment of COVID-19 of the RCTs noted above are a handful of prominently cited RCTs of this type with negative findings, including TOGETHER-IVM, three others coauthored by Boulware [33,34,35], and a fourth published in 2021 [36] which had several major protocol violations [37]. In that 2021 RCT, IVM was mistakenly substituted for placebo doses for 38 patients and blinding was broken by the study’s use of sugar water as the placebo for one-third of the patients (liquid IVM is bitter). Adverse events that are distinctive to the high IVM dose used (transient and non-critical) occurred at almost identical rates in the IVM and placebo arms, while over-the-counter (OTC) sales of IVM surged in the study region during the study period [37]. Indeed, IVM use for COVID-19 in Latin America was so extensive in 2020 that it was found to be difficult to test there in clinical trials [38].

Although RCT results, which are mixed for IVM treatment of COVID-19, as noted, are generally required to draw conclusions about therapeutic efficacy, certain drugs such as penicillin were successfully deployed based upon clear evidence of curative results and corresponding in vitro activity that predated unequivocal RCT findings. Such success was also achieved, for example, using antibiotics and bismuth to treat peptic ulcers, a condition for which H2 receptor antagonists such as Tagamet and Zantac (the latter recalled in 2020 due to carcinogenic content [39,40]) yielded palliative but rarely curative benefits [41,42]. Following initial indications of efficacy for this combination therapy [43,44], an uncontrolled clinical trial conducted in Australia in 1990 by Thomas Borody reported 96% curative results for peptic ulcers using tetracycline, metronidazole and colloidal bismuth administered over four weeks [45]. Although clear RCT evidence of efficacy for that triple therapy was not amassed until 1992 [42], in Australia such combination treatments of peptic ulcers began to be used widely in the late 1980s, with a sharp drop in associated mortality beginning in 1990 and an estimated 18,665 deaths prevented between 1990 and 2015 [46]. However, this triple-therapy cure was not widely used in the rest of the world until the late 1990s, after the patents for Tagamet and Zantac expired [47]. This treatment is now the worldwide standard of care for peptic ulcers, with the associated discovery of its bacterial cause honored with the Nobel Prize for Medicine in 2005.

In 2022, the Australian researcher Thomas Borody collaborated in one of three clinical studies [48] which each found sharp increases in peripheral oxygen saturation (SpO2) for severe COVID-19 patients within 24 h after treatment with IVM [48,49,50]. The biochemical mechanism that appears to explain this rapid normalization of SpO2 is competitive inhibition by IVM of glycan bindings to spike protein (SP) of SARS-CoV-2 [51]. These glycan bindings as manifested, for example, by virally induced hemagglutination, are critical to host cell attachments and the morbidity of betacoronaviruses; the two non-lethal strains, HKU1 and OC43, of the five human betacoronaviruses express an enzyme that likewise abrogates these glycan bindings [52]. A recent study demonstrated that SARS-CoV-2 SP mixed with human red blood cells caused hemagglutination and that IVM blocked that hemagglutination and reversed it after it formed [53]. If this in vitro study is successfully replicated and extended in vivo, then, with a clear biochemical foundation, the three SpO2 tracking studies for IVM administration noted could constitute a meaningful demonstration of IVM efficacy against COVID-19, in the face of mixed RCT findings. In such an eventuality, it would be important to appreciate that TOGETHER-IVM, pending disclosure of its PP outcomes, adds no credible contribution to this mix of evidence.

## 4. Questions Concerning Scientific Integrity in the Three RCTs Considered

COI disclosures show that coauthors of the Bretthauer et al. 2022 colonoscopy study [6] and the companion editorial [7] collectively had financial relationships, mainly consulting or speaker fees, with nine manufacturers of advanced CRC detection equipment [54]. The potential boost to sales of such equipment that could arise from underreported benefits of routine colonoscopy screenings raises some concern about these COIs.

Of interest regarding the second and third RCTs of our focus, which tested FLV and IVM for treatment of COVID-19, is a history of bias against generic drugs in favor of patented medicines, exploiting the vulnerability of science to commodification and regulatory capture [55,56]. The delay of almost a decade in the worldwide deployment of the triple therapy for peptic ulcers until after patents for the two best-selling palliative drugs for that condition expired, as described above, is a notable example. Some possibility of such a bias against IVM was raised in a 4 February 2021 press release from Merck, which was then developing the COVID-19 therapeutic molnupiravir, which claimed there was “a concerning lack of safety data” for IVM [57]. Yet IVM is Merck’s own drug, and the Nobel Prize committee specifically noted IVM’s safety record in honoring the discovery of this drug in its 2015 prize for medicine [58]. IVM has been used safely in 3.7 billion human doses worldwide since 1987 [22] and been proven safe at much-higher-than-standard doses [59,60]. Major media reports of IVM poisonings emerged in the wake of this Merck press release, which were later debunked [57]. Having been used for COVID-19 treatment in 25 countries by the end of 2020 [30], of all generic treatments for this disease, IVM presented the greatest competitive jeopardy to patented COVID-19 therapeutic offerings.

Certain actions and communications by coauthors of TOGETHER-IVM do not appear compatible with a commitment to data transparency, an ethical norm that serves to preserve trust in the reporting of clinical trials. Their misdirection to a non-existent ICODA database in that study’s DSS and in an email response to the 10 May 2022 letter requesting PP outcomes for deaths and hospitalizations certainly raises concern. Notable cases of fabricated clinical trial outcomes in the past decade have been uncovered after failure by investigators to provide their studies’ underlying data when asked [61,62]. For example, in 2020, Sapan Desai, a Surgisphere-affiliated coauthor of studies in the Lancet [63] and in the New England Journal of Medicine (NEJM) [64], was queried about anomalies in the Lancet study but refused to provide the underlying data for review. Those studies and another coauthored by Desai were then retracted within a few weeks [65,66,67,68].

In addition, after failing to disclose PP outcome numbers for deaths and hospitalizations, as first requested by email on 11 April 2022, Edward Mills, a coauthor of all four TOGETHER trial publications, instead suggested to the questioner that she inform her colleagues that “the world is not flat” (Appendix C, item 7). When asked by Reuters on 22 November 2022 about USD 18 million in funding commitments for the TOGETHER trial by FTX, the scandal-plagued cryptocurrency firm, Mills replied by email that “no funding was received (from FTX) prior to May 2022”, when in fact FTX was listed as a TOGETHER trial funder on the latter’s website by 3 March 2022 (Appendix C, item 8). Another obstacle to full transparency regarding TOGETHER-IVM was that the journal in which it appeared, the NEJM, failed to publish any of the many letters submitted about it (Appendix C, item 9).

It would seem that prompt corrective action would have followed after an ICODA manager asked the TOGETHER-IVM coauthors, as she reported on 7 June 2022, to stop listing ICODA in their DSS and those coauthors were then reminded twice by email on 5 June and 11 July that ICODA never held TOGETHER trial data (Appendix C, item 10). The overlapping group of mostly the same TOGETHER trial coauthors could have promptly changed the DSS for all four of their publications. Yet as of 22 May 2023, the online versions of TOGETHER-FLV, published on 27 October 2021, and of another TOGETHER trial publication of 13 December 2021 [14] still list their data sources as ICODA [26]. TOGETHER-FLV, in fact, updated its DSS on 9 August 2022, to still declare that the study data was on ICODA but to add that “other study related documents can be found in Vivli.” Yet as noted, ICODA never held any TOGETHER trial study data, and as of 22 May 2023, Vivli shows “no files” in the study documents tab for the TOGETHER trial [26].

## 5. Discussion

In drawing conclusions about ITT vs. PP outcomes for the three RCTs of our focus, it is important to reiterate that ITT analysis has been the preferred approach for nonequivalence trials since the “healthy adherers” effect became apparent in a major RCT for coronary heart disease drugs in 1975 [11,69,70]. Although PP analysis is subject to several possible sources of bias [1,11,71], ITT analysis can have other potential distortions [72], which can be of particular concern when protocol compliance is suboptimal [1,3,4,5]. A judgment as to which of these analytical methods would likely reflect real-world efficacy of the intervention being tested in a given RCT will depend upon details of clinical trial design and the extent and characteristics of non-compliers in both the treatment and placebo arms. Yet for the first two RCTs of our focus, the investigators themselves, as quoted above, asserted that PP analysis was most likely to reflect the efficacy, respectively, of the intervention and drug tested. Although these judgments are not unimpeachable, they presumably reflect the knowledge of the underlying complexities of the clinical trial design and the characteristics of noncompliers, potentially requiring a review of unpublished de-identified patient data, by their respective teams of investigators.

In addition, in view of the sizable extent of protocol noncompliance in the placebo arm of the third RCT of our focus, the requirement stated by Porta et al., 2007, is sound: “In the presence of protocol deviations, the conclusion of a CT cannot rest on the single reporting of either the ITT or the PP approach alone” [4]. The authors of TOGETHER-IVM may be able to provide fully convincing arguments for the real-world relevance of their reported ITT outcomes. However, to withhold reporting of the four requested PP outcomes for deaths and hospitalizations, treatment vs. matching placebo, and to misdirect investigators to a non-existent database in response to requests for underlying data, does not allow either the investigators or the scientific community to justify or question any such claims.

The first two RCTs of our focus, the 2022 colonoscopy study and TOGETHER-FLV, both provide examples of how ITT and PP analysis can provide sharply different indications of the efficacy of a medical intervention when per-protocol compliance is suboptimal. For the first example, the PP outcome—a 50% reduction in CRC deaths among the 42% of participants in the intervention group who actually underwent the colonoscopy screening procedure—is the statistic of relevance for someone considering whether to undergo this procedure, and for public health. The study coauthors themselves stated that this PP outcome likely defined the medical efficacy of colonoscopy screenings, yet the study report and an accompanying editorial emphasized ITT findings of only minor benefits.

For the second RCT considered, TOGETHER-FLV, PP analysis found a ten-fold reduction in deaths, FLV treatment vs. placebo (RR = 0.09, *p* = 0.022), whereas ITT analysis found a more limited 31% reduction in deaths (RR = 0.69, *p* = 0.24). PP analysis indicated that FLV might have “considerable clinical benefits” against COVID-19, the study paper concluded, while a researcher closely associated with the TOGETHER-FLV team “relied heavily on the PP analyses,” and a senior author of TOGETHER-FLV stated that the study found FLV “potentially provides a very large treatment effect,” a conclusion supported by its PP analysis, not by its ITT analysis. This study thus demonstrates how ITT and PP analyses of a treatment effect can differ widely, even with suboptimal but not outsize extents of protocol noncompliance (with 74% and 82% protocol compliance, respectively, for treatment and placebo), and how in such cases PP analysis may best reflect real-world clinical benefits.

For TOGETHER-IVM, PP outcomes were needed to draw reliable conclusions about drug efficacy, since protocol noncompliance in the placebo group was larger than that for TOGETHER-FLV and confounded by inconsistent reporting. As detailed in Appendix B, the TOGETHER-IVM study paper and other study documents provided three conflicting descriptions of the ITT placebo group. Defying any usual meaning of per-protocol, the TOGETHER-IVM study paper states at different points that its ITT placebo group included mixed placebos (either 3-day and 14-day only, or 1, 3, 10 and 14-day), but that the PP placebo group dropped the mixed placebos, to include only the 3-day placebos. No rationale for this unusual selection from the ITT-to-PP placebo group is provided, and no other TOGETHER trial publication mentions excluding mixed-day placebos from the PP placebo group.

Furthermore, the use of mixed-day placebos violates a core principle of RCTs, that of having a placebo arm that is blinded and matched to the drug arm. As the NIH noted for TOGETHER-FLV, but which is applicable to all TOGETHER trial arms, a “key limitation” of its adaptive platform trial design was that “not all patients in the placebo arm received a placebo that was matched to fluvoxamine by route of administration, dosing frequency, or duration of therapy” [17]. One-day placebo patients for TOGETHER-IVM, as study documents repeatedly report as being included in the placebo group, would include those receiving a single mock injection, thus clearly not blinded (Appendix B). PP numbers for the TOGETHER-IVM placebo group, 3-day placebos only, as specified in Table 1, matching the 3-day IVM treatment group, must therefore be disclosed. These PP numbers are needed to overcome the NIH’s noted limitations of the TOGETHER trial’s adaptive platform trial design [17], to penetrate this maze of inconsistencies and allow useful conclusions to be drawn.

In addition, potential use of IVM by placebo patients in the TOGETHER trial, possibly based upon different markings and taste of the IVM vs. placebo tablets or on other violations of blinding noted, may have compromised the study’s findings. OTC IVM sales were in fact extensive in the TOGETHER trial’s Brazilian locale during its IVM study period [73]. If part of the reduction in the placebo group from ITT to PP was due to placebo patients surreptitiously taking OTC IVM and becoming lax in protocol compliance, then the ITT analysis could have been biased against finding IVM to be effective, if indeed IVM is effective.

If the four PP outcome numbers for deaths and hospitalizations, IVM treatment vs. placebo, had supported TOGETHER-IVM’s negative conclusion on IVM efficacy for COVID-19, it would appear that study coauthors would have hastened to disclose them. Edward Mills, a senior author of that study, had reported that negative conclusion for IVM efficacy eight months before the study’s publication, in a video presentation of 6 August 2021, in which he described an adversarial relationship between some IVM proponents and the TOGETHER trial team [74] (at minutes 31 and 49). PP outcomes consistent with ITT outcomes would have bolstered his case. The failure of study coauthors to disclose the four PP outcome numbers of key interest for deaths and hospitalizations, IVM treatment vs. matching 3-day placebo, in response to six email requests, and the misdirection instead to a non-existent ICODA database, do not inspire confidence in either that group’s commitment to data transparency or in the study’s reported negative conclusion on IVM efficacy against COVID-19. Disclosure of these PP outcome numbers and supporting data summarized in Table 1, as requested on 19 July 2022 [24], would allow this study to be credibly considered in the mix of evidence for COVID-19 treatment using IVM.

## 6. Conclusions

Three examples of RCTs were considered for which ITT and PP outcomes were either known or indicated to be substantially different. In the first case, PP analysis reflected a 50% reduction in CRC deaths from colonoscopy screenings as performed, which according to the study coauthors themselves defined the screening’s medical efficacy. Its ITT analysis, in contrast, reflected minimal benefits provided, with only 42% of the intervention group undergoing this procedure. In the second case, coauthors of the TOGETHER-FLV study found the ten-fold reduction in mortality as calculated by PP analysis more predictive of real-world benefits of FLV than the lesser, statistically insignificant reduction in mortality of its ITT outcome. In the first and second cases, all PP outcomes were reported, which differed sharply from the ITT outcomes, and the investigators of the respective RCTs themselves found that the PP outcomes were most representative of medical efficacy.

For the third example, TOGETHER-IVM, however, the investigators refused to report the study’s PP outcomes which were of key importance, those for death and hospitalization rates, IVM treatment vs. matching placebo, despite repeated requests to disclose these figures. Instead, they misdirected the scientific community to a data repository which never held the study’s data. These PP outcomes for this study, like those of the first two RCTs considered, may differ significantly from its ITC outcomes, and may be most representative of actual drug efficacy. These four PP outcome numbers of key interest must be disclosed, especially in view of inconsistencies and irregularities in the reporting of protocol compliance for the study’s placebo group, which call its ITT outcomes into question. Reliable conclusions about this study’s findings cannot otherwise be credibly drawn.

## Figures and Tables

**Table 1:** Data requested: outcome numbers (top) and fields for deidentified patient data (bottom).

Individual Data Outcome Values		Ivermectin Treatment	Placebo			
			1-Day	3-Day	10-Day	14-Day
Number of patients		679	**0?**	679?	**0?**	**0?**
Number of patients, PP		624	**0?**	288?	**0?**	**0?**
Number of deaths, PP		➖	**0?**	➖	**0?**	**0?**
Number of hospitalizations, PP		➖	**0?**	➖	**0?**	**0?**
The four values denoted by dashes have been requested. Note that red zeroes denote values which were not specified, but indicated to be zero at several points in the TOGETHER-IVM study paper and associated documents (see Appendix B, item 1 of this paper). If any of those values are not in fact zero, please fill them in and adjust other values in the placebo block accordingly.						
Pt. ID	Age Group	Sex	Treatment or Placebo; If Placebo, Enter 1, 3, 10 or 14 For Days of Placebo Use	Complied with Protocol (Y, N)?	Mortality Outcome (Died, Survived, Unknown)	Hospitalization Outcome (Hospitalized, Not Hospitalized, Unknown)
➖	➖	➖	➖	➖	➖	➖

## Data Availability

Not applicable.

## Abbreviations

The following abbreviations are used in this manuscript:

COI	conflict of interest
COVID-19	coronavirus disease 2019
DSS	data sharing statement
FLV	fluvoxamine
ITT	intention-to-treat
IVM	ivermectin
OR	odds ratio
OTC	over-the-counter
PP	per-protocol
RCT	randomized clinical trial
RR	relative risk
SARS-CoV-2	severe acute respiratory syndrome coronavirus 2
SP	spike protein
SpO2	peripheral oxygen saturation

## Appendix A. Timeline of Denial of Access to De-identified Patient Data for TOGETHER-IVM

**30 March 2022.** The DSS of the study as published on this date promised access to its “complete de-identified data set” at the ICODA data repository beginning “immediately after publication” [26]. **10 May 2022.** A letter from 66 scientists and physicians requesting the study’s four outcome numbers of key importance, PP death and hospitalization rates, IVM vs. placebo, was emailed to study coauthors, and copied to 39 scientists and science journalists [24]. **10 May 2022.** TOGETHER trial coauthor David Boulware replied, declining to provide those outcome numbers and directing the letter signatories to the ICODA data repository [25]. **7 June 2022**. An ICODA manager sent an email to a scientist who had inquired about this data, stating “ICODA does not hold the data and we have requested that the authors update the data sharing agreement to reflect that” [28]. **8 June 2022**. Three of us (DES, CA, EJF) emailed the TOGETHER trial coauthors and cc’s of the above-cited email thread, quoting the above statement from that ICODA manager, that ICODA never hosted the study’s data [24]. The study’s DSS continued to specify the same link to ICODA through 6 July 2022. **7 July 2022**. The study’s DSS was changed to link to another data repository, available online: vivli.org (accessed on 20 March 2023) [26]. **14 July 2022**. An entry for the TOGETHER platform trial appeared on Vivli, matching the NCT number (NCT04727424) for the TOGETHER-IVM study [26]. This vivli.org record, for its “intervention/treatment,” listed three treatment drugs other than IVM, but not IVM [75]. The ClinicalTrials.gov record for the TOGETHER trial, v4, (Available online: https://clinicaltrials.gov/ct2/history/NCT04727424?V_4 (accessed on 15 January 2022)), and also v5, (Available online: https://clinicaltrials.gov/ct2/history/NCT04727424?V_5 (accessed on 3 July 2022)), lists those same three drugs but not IVM as the treatment agents. **19 July 2022**. Three coauthors of the current analysis (DES, CA, EJF) emailed study coauthors noting this omission of IVM in the vivli.org record for the TOGETHER trial [24]. **21 July 2022**. This vivli.org record was changed to include IVM [26]. **Vivli’s legal agreement for data access** [76], p. 1, references the option for an “alternate review model approved by the relevant Data Contributor,” i.e., the study coauthors, to govern whether an application for data access is approved or rejected. **That Vivli legal agreement** also includes the provision for unspecified conditions to be imposed by the study author (“data provider”) and/or Vivli upon use of the data [76], p. 2, Section 1b: “Recipient agrees to comply with any conditions that were placed by the cognizant Data Review Entity(ies) [the data provider and/or Vivli] on Recipient’s use of the Data Sets.” With such latitude for unspecified conditions to be imposed, these could, for example, limit the disclosure of a potential finding of manipulated results.

## Appendix B. Conflicting Descriptions of the TOGETHER-IVM Placebo Group

1. **Repeated references indicating that only a 3-day placebo group was used for analysis** - In an NEJM twitter thread of 30 March 2022, when asked about the composition of the placebo group of TOGETHER-IVM, coauthor David Boulware replied: “The analysis is via concurrently randomized placebo only. Stated a few places, including in Figure 1 caption. ‘Only the results in the 3-day ivermectin group as compared with the concurrent placebo group are reported in this article’” [77]. (Figure 1 to which Boulware refers is from the TOGETHER-IVM study paper [15]). - In response to a follow-up query, “placebo same 3 days?” Boulware replied: “…in this case, yes a 3d [3-day] placebo was run while IVM was running” (as cited above). - The TOGETHER-IVM study protocol, p. 169, Appendix 1.2, ivermectin, specifies: “Placebo administration: Once daily for 3 days”. 2. **A vague and inconsistent mixed-day placebo scenario** - The TOGETHER-IVM study report, p. 5, states that 3-day and 14-day placebos were included in the placebo group: “Although all the participants who had been assigned to the 3-day and 14-day placebo regimens were included in the intention-to-treat population, only those who had been assigned to the 3-day placebo regimen were included in the PP population”. - However, that study report also has references to 1-, 3-, 10- and 14-day placebos scattered throughout the study paper, e.g., for Table 3 (of [15]), p. 9: “The duration of placebo use was 1, 3, 10, or 14 days.” - Its caption for Figure 1, p. 6, states: “Participants in the placebo group received placebo for 1, 3, 10, or 14 days,” but the caption also states: “only the results in the 3-day ivermectin group as compared with the concurrent placebo group are reported in this article”. - The 1-day placebo for the TOGETHER trial that corresponded to its interferon lambda treatment was given by injection, as was the drug (the ClinicalTrials.gov record for the TOGETHER trial, v5, available online: https://clinicaltrials.gov/ct2/history/NCT04727424?V_5 (accessed on 3 July 2022)). Interferon lambda was administered to 69% of the patients in the TOGETHER trial who took a 1-day drug course (TOGETHER-IVM, p. 6). A 3-day oral treatment course of IVM could hardly be blinded using a 1-day placebo consisting of a single placebo injection. - The study does not specify numbers of patients for any of these mixed-day placebos, either for 1-, 10- or 14-day, or for only 14-day, placebos. - Without any stated or apparent rationale, the TOGETHER-IVM study report, Figure 1 caption, p. 6, states that whereas 1-, 3-, 10- and 14-day placebos were included in the study’s ITT group, ”only those in the 3-day placebo groups were included in the PP population.” This is a discrepant variation of the above-quoted assertion that the ITT placebo group consisted of 3-day and 14-day placebo patients, but “only those who had been assigned to the 3-day placebo regimen were included in the PP population”. - In the study report and supplementary documents of TOGETHER-IVM’s three sister publications for other drug arms, nowhere is any such drop-out of mixed-day placebos described for the PP placebo group [12,13,14].

## Appendix C. Additional Notes

1. Hernan and Robins stated that the rate of protocol compliance in Holme et al. 2014 was 70% [3], but Holme et al. reported in their publication that rate as 63% [10]. Note that Bretthauer et al., 2022 [6] had five coauthors in common with Holme et al. 2. The only very large treatment effect of TOGETHER-FLV was its ten-fold reduction in mortality by PP analysis. No ITT measure of efficacy, except for reduction in emergency room visits, even achieved the threshold of *p* < 0.05 for statistical significance. 3. As detailed in Appendix B, conflicting descriptions of the TOGETHER-IVM placebo group were provided in its study paper and other study documents. These describe TOGETHER-IVM’s ITT placebo group as consisting either of 3-day and 14-day placebo patients (matching the TOGETHER trial treatment arms having the corresponding numbers of days of drug delivery); of 1-, 3-, 10- and 14-day placebo patients; or of 3-day placebo patients only. Defying any usual meaning of per-protocol, the TOGETHER-IVM study paper stated at different points that its ITT placebo group included mixed placebos (either 3-day and 14-day only or 1-, 3-, 10- and 14-day), but that the PP placebo group dropped the mixed placebos to include only 3-day placebos. No rationale for this unusual selection from the ITT to PP placebo group or breakdown of any of these variously described mixed-day placebos was specified. Furthermore, none of the other three TOGETHER trial publications mentioned excluding mixed-day placebos from the PP placebo group. The different values for the placebo ITT-to-PP decrease in Tables 2 and 3 (of the study, [15]) arise from these conflicting explanations. 4. The 10 May letter and four follow-up emails [24] were addressed to 11 coauthors of TOGETHER-IVM (those whose email addresses could be identified), five members of its data and safety monitoring committee and the editor-in-chief of the journal that published this study (the NEJM), and copied to 39 science journalists, bioethicists and other scientists. 5. IVM was also omitted in the ClinicalTrials.gov record for the TOGETHER trial in its versions of both 15 January 2022 and 3 July 2022 (see Appendix A, above). On 19 July 2022, three coauthors of this analysis (DES, CA, EJF) emailed the TOGETHER-IVM coauthors about the omission of IVM. Two days later, the Vivli record was changed to include it among its “intervention/treatment” agents [24]. The Vivli data have two additional major shortcomings, listed in Appendix A. 6. The mortality rates were ≤ 4% in both the treatment and placebo groups for all but one of the ten studies included in this June 2022 meta-analysis, and therefore relative risk (RR) was very close to the odds ratio (OR), with the pooled value of the latter = 0.51. The mortality reduction for the RCTs rated as having a low risk of bias was less (log OR = −0.12), but the TOGETHER-IVM study was included in that low-risk-of-bias group, weighted to account for 63% of its pooled log OR. 7. To the first of the emails requesting PP outcomes for deaths and hospitalizations sent on 11 April 2022 by one of us, Colleen Aldous, study coauthor Edward Mills did not disclose those outcome numbers, but answered another question; Aldous thanked him, and said she would share his response with other investigators. Mills replied “Do. You may want to also let them know the world is not flat” [23]. 8. The dating of the FTX funding was of interest to Reuters with respect to whether it was in place during the TOGETHER trial’s evaluation of IVM [78]. As determined from web archive records [79], FTX funding for the TOGETHER trial first appeared on the latter’s website sometime after 3 December 2021 and before 3 March 2022. The first FTX grant to the TOGETHER trial was in the amount of USD 3.25 million, followed by a second, USD 15 million FTX grant awarded in May 2022 [80]. After the May grant was announced, “Funded by FTX” and the FTX logo appeared near the top of the home page of the TOGETHER trial’s website, as shown in archival website copies between 12 May and 19 November 2022. All references to FTX funding of the TOGETHER trial were dropped from the latter’s website within a few days before 22 November 2022, the date of the Reuters report [79]. 9. This failure to publish any of these submitted letters may be reflective of editorials that the NEJM published in 2015 and 2016, which downplayed concerns about data sharing and conflicts of interests [81,82,83,84,85]. These editorials prompted sharp dissent by three former senior editors of the NEJM [86] and others [87]. The NEJM also published the Bretthauer et al., 2022 colonoscopy study and accompanying editorial. 10. On 7 June, an ICODA manager emailed one of the scientists who had inquired about this data, stating: “ICODA does not hold the data and we have requested that the authors update the data sharing agreement to reflect that” [28]. These follow-up emails, which noted that ICODA never held TOGETHER trial data, were sent to recipients including 11 TOGETHER-IVM coauthors and 39 cc’s [24], as described in item 3 above.
